# A Natural Bone Cement—A Laboratory Novelty Led to the Development of Revolutionary New Biomaterials

**DOI:** 10.6028/jres.106.053

**Published:** 2001-12-01

**Authors:** Laurence C. Chow, Shozo Takagi

**Affiliations:** American Dental Association Health Foundation, Paffenbarger Research Center, National Institute of Standards and Technology, Gaithersburg, MD 20899-0001

**Keywords:** biomaterial, bone graft, bone repair, dicalcium phosphate anhydrous, hydroxyapatite, self-setting calcium phosphate cement, tetracalcium phosphate

## Abstract

Research on calcium phosphate chemistry at NIST led to the discovery of the worlds first self-hardening calcium phosphate cements (CPC) in 1987. Laboratory, animal, and clinical studies were conducted to develop CPC into clinically useful biomaterials. The combination of self-hardening capability and high biocompatibility makes CPC a unique material for repairing bone defects. Near perfect adaptation of the cement to the tissue surfaces in a defect, and a gradual resorption followed by new bone formation are some of the other distinctive advantages of this biomaterial. In 1996 a CPC, consisting of tetracalcium phosphate and dicalcium phosphate anhydrous, was approved by the Food and Drug Administration (FDA) for repairing cranial defects in humans, thus becoming the first material of its kind available for clinical use. This paper will review the course of the development, the physical and chemical properties, and clinical applications of CPC.

## 1. Introduction

In the early 1980s, Brown and Chow of the American Dental Association Health Foundation Paffenbarger Research Center (ADAHF-PRC) at the National Institute of Standards and Technology (NIST) conducted studies on calcium phosphates aimed at developing remineralizing pastes to repair early dental carious lesions. Based on the solubility properties of calcium phosphates [1], they formulated mixtures containing tetracalcium phosphate (TTCP), Ca_4_(PO_4_)_2_O, and dicalcium phosphate anhydrous (DCPA), CaHPO_4_, or TTCP and dicalcium phosphate dihydrate. When mixed with water these mixtures would rapidly form hydroxyapatite (HA), Ca_5_(PO_4_)_3_OH, which is the major component of tooth mineral, at body temperatures. It was observed that some of the pastes became a hardened mass when left in test tubes for a few hours. Thus, the PRC scientists inadvertently discovered a new type of self-hardening cement that consisted of only calcium phosphates and formed HA as the only product.

Since Brown and Chow reported the first CPCs in 1987 [2], many different combinations of calcium and phosphate-containing compounds have been investigated as potential CPC materials and the properties of the various cements were studied. A large number of publications on CPC have appeared in the last several years, which have been summarized in [3] and [4], reflecting a sharp increase in the interest in CPC research. This paper provides a brief review of the chemistry, properties and applications of the CPC based on TTCP + DCPA.

## 2. Chemistry of Setting Reaction of CPC

**Fig. 1:**
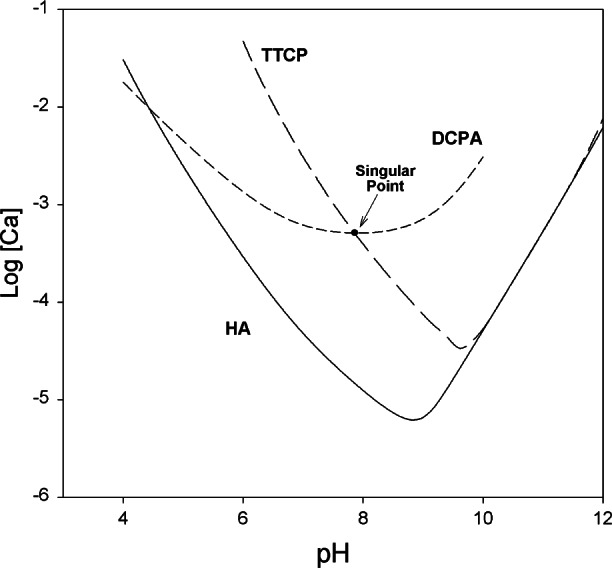
Solubility phase diagram for the ternary system, Ca(OH)_2_–H_3_PO_4_–H_2_O, at 25 °C showing solubility isotherms of TTCP, DCPA and HA.

The setting reactions of CPC may be understood by analyzing the solubility of the individual cement components such as TTCP and DCPA. The solubility phase diagram showing the solubility curves of TTCP, DCPA and HA is given in Fig. 1. Each curve describes the solubility of a salt, expressed as the calcium concentration of the saturated solution as a function of pH. At a given pH, a salt whose isotherm lies below that of another salt is less soluble (thermodynamically more stable) than the other. The point where two isotherms cross is known as a singular point. The solution at the singular point is saturated with respect to both salts, which are equally stable at this solution composition. The fact that HA is the least soluble salt over a wide range of pH explains why the major mineral component of both normal hard tissues and pathological calcified deposits are impure forms of HA. When the pH falls below approximately 4.2, DCPA becomes the least soluble. TTCP is the most soluble salt at pH below 9. The relative stability of these two salts is the major driving force for the setting reactions that occur in calcium phosphate cements. Generally, both TTCP and DCPA are considerably more soluble (less stable) than HA at pH above 4.2. As a result, a solution saturated with respect to TTCP (or DCPA) is supersaturated with respect to HA. This would lead to HA precipitation and the major driving force for the CPC setting, which, in turn, would make the solution undersaturated with respect to TTCP and cause more TTCP to dissolve to re-attain saturation. Thus, it is the relative solubility of the various salts that provides the driving force for the setting reactions that occur in CPC.

In addition to being highly soluble in neutral and acid pHs, TTCP is the only calcium phosphate salt that has a Ca/P ratio higher than that of HA. Thus, it plays a special role as a cement component in that only TTCP can be combined with other calcium phosphate salts with lower Ca/P ratios such as DCPA to formulate a mixture that has the stoichiometry of HA. Formation of HA in such a mixture, given by Eq. (1), does not release acidic or basic by-products.

$${\text{Ca}}_{4}{\left({\text{PO}}_{4}\right)}_{2}O+{\text{CaHPO}}_{4}\to {\text{Ca}}_{5}{\left({\text{PO}}_{4}\right)}_{3}\text{OH}$$ (1)

As long as both DCPA and TTCP are present in excess and the rates of dissolution of these two salts are greater than the rate of HA formation, the solution composition would remain near or at the singular point for these two salts (Fig. 1) and the conversion of DCPA and TTCP to HA would continue. Thus, the liquid phase of the cement would remain at a near constant pH and composition, allowing the HA formation to proceed at a steady rate [5]. The singular point pH for the TTCP + DCPA is approximately 8. Thus, during the HA formation, the pH of the cement would only be slightly higher than the physiological pH, and this may contribute to the high biocompatibility observed for CPC.

Setting of the TTCP + DCPA cement with water as the liquid phase occurred in about 30 min at 37 °C, and the conversion of the starting materials to HA was almost completed in 4 h [5]. During this period, the cement setting reaction proceeded at a near-constant rate, suggesting that the reaction rate was limited by factors that are unrelated to the amounts of the starting materials and the reaction products present in the system. Such factors could be, for example, the particle sizes of DCPA or TTCP.

## 3. CPC Materials

A CPC usually consists of a powder and a liquid phase. The powder phase is an equimolar mixture of TTCP and DCPA or mass fractions of 0.729 of TTCP and 0.271 of DCPA. TTCP is prepared by heating a mixture of commercially available DCPA^1^ (Baker Analytical Reagents, J. T. Baker Chemical Co., NJ, U.S.A.) and calcium carbonate, CaCO_3_, (J. T. Baker Chemical Co.) at 1500 °C for 6 h in a furnace [Eq. (2)], followed by quenching at room temperature in a desiccator [6].

$$2{\text{CaHPO}}_{4}+2{\text{CaCO}}_{3}\to {\text{Ca}}_{4}{\left({\text{PO}}_{4}\right)}_{2}O+H_{2}O+2{\text{CO}}_{2}↑$$ (2)

Because the particle size of the cement ingredients plays an important role in the setting and final properties of the cement, TTCP is ground to a median particle size of approximately 17 μm and commercial reagent grade DCPA is ground to a median particle size of about 1 μm. It is proven essential to grind each solid component individually to the desired particle sizes [1]. The two components are then thoroughly mixed and the resulting CPC powder is stored in a desiccator.

The liquid phase can be water, saline or other physiological fluids. A phosphate containing solution is used as the liquid for a shorter hardening time of 5 min [7]. For applications requiring longer working times, a non-aqueous but water-miscible liquid such as glycerine [8] or polyethylene glycol [9] can be used. Ready-to-use premixed CPC pastes are also prepared with these non-aqueous liquids [10]. Because the cement setting reaction does not occur to a significant extent in these liquids, the cement would remain pliable for an indefinite period and harden after being placed in a defect and a sufficient amount of water from the surrounding tissues diffused into the paste. Addition of a jelling agent such as hydroxypropyl methlycellulose, carboxyl methylcellulose, chitosan, etc., to the cement liquid can improve the washout resistance of the cement paste before hardening occurs, allowing CPC to harden in standing water [11]. Chitosan is also be used to prepare CPC materials that are less brittle and non-rigid [12, 13].

## 4. Physical and Chemical Properties of CPC

**Fig. 2:**
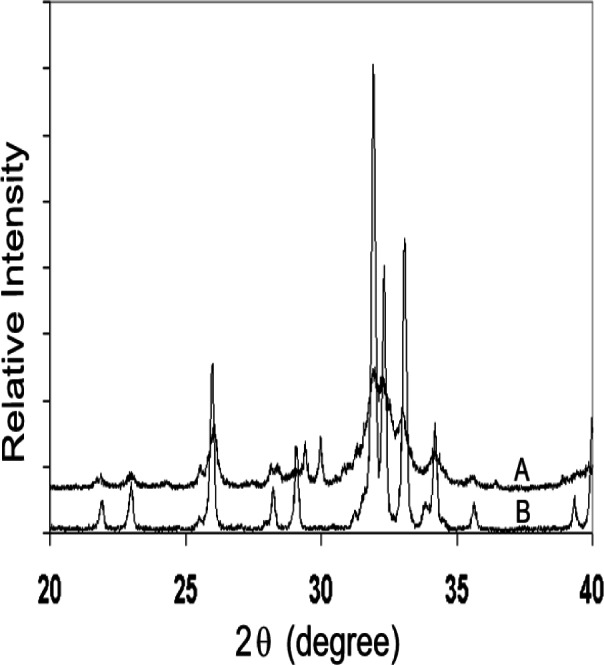
X-ray patterns of (A) 24 h set CPC sample and (B) Ceramic HA showing poorly crystalline HA formation in the CPC.

**Fig. 3:**
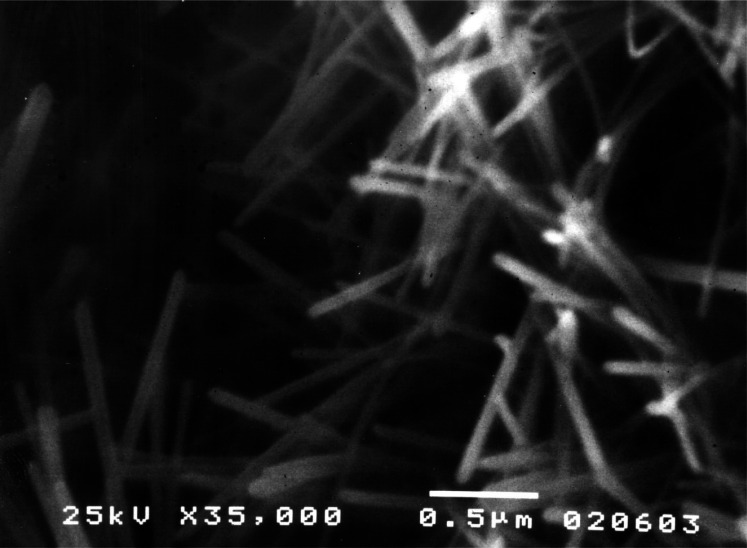
SEM micrograph of a fractured surface of 24 h set CPC sample showing rod-like crystallites.

A CPC with a setting time of about 30 min with water and a compressive strength of 34 MPa was developed [5]. Powder X-ray diffraction (Rigaku DMAX 2200, Rigaku/USA, MA) and microscopic examinations by scanning electron microscopy (JEOL JSM-5300, JEOL USA Inc., MA) indicated that the HA formed in CPC (Fig. 2) was in the form of very small rod-like crystals (≈0.05 μm × 0.5 μm) (Fig. 3) that were similar in size to the HA crystallites in human tooth enamel. Continued research conducted by ADAHF-PRC scientists at NIST has resulted in many significant improvements in CPC. These improvements included a much shorter hardening time of 5 min [7] using a phosphate containing solution as the cement liquid, a considerably higher diametral tensile strength and compressive strength of 11.5 MPa and 66 MPa, respectively [14]. Additional improvements included increased washout resistance, premixed CPC pastes and non-rigid CPC, as described above [10 to 13].

Solubility is an important property of CPC because it relates to the stability of the material under various application conditions. Fully cured CPC samples prepared from the TTCP + DCPA mixture contain essentially HA with a small amount of residual TTCP (Fig. 2) and have solubility properties similar to those of HA. CPC is nearly insoluble in water but is readily soluble under strong acidic conditions. In vivo, CPC is insoluble under normal physiological conditions because fluids such as saliva and blood are supersaturated with respect to HA. However, CPC dissolves under acidic conditions created by osteoclasts and other acid-producing cells.

## 5. In Vivo Characteristics of CPC

CPCs contain only calcium phosphates and form apatitic mineral as product upon setting at neutral pH. Consequently, they were found to be highly compatible with both hard and soft tissues. In numerous animal studies [15 to 24], CPCs have proven superior to currently available materials, due to their ability to harden at the application site. Applied as a paste, CPC closely adapts to bone surfaces even when examined at the microscopic level. Direct and close adherence of CPC to bone reduces the formation of intervening tissues at the bone-implant interface, facilitating the processes that lead to replacement of CPC by new bone.

In addition to high biocompatibility and self-hardening, the usefulness of CPC for repairing bone defects arises from its unique in vivo properties: gradual resorption and replacement by new bone formation with no loss in volume. Set CPC consists of tightly packed microcrystalline HA that has a large surface area. Because HA is formed in an aqueous environment and has a relatively low crystallinity (Fig. 2), it is similar to biological apatite. These properties are believed to be responsible for CPC’s in vivo resorption characteristics. This is in contrast to the ceramic HA materials, which are non-resorbable [25], or β-tricalcium phosphate, β -Ca_3_(PO_4_)_2_, which resorbs but is not always fully replaced by new bone formation [26].

## 6. Clinical Studies of CPC

The TTCP + DCPA cement, shown to be efficacious in animal studies, was evaluated in several medical centers for cranial defect repair as part of a U.S. FDA approved study in human subjects. From eleven neurotologic procedures completed in 1994, it appeared that the cement had potential to become a commonly used material in the management of cranial base and temporal bone defects following surgery [27]. Suboccipital craniectomy defects resulting from vestibular schwannoma removal were reconstructed by CPC [28]. Within 2 years, re-establishment of cranial bone integrity was reported. Cranial base reconstruction with CPCs has been successful for translabyrinthine, middle cranial fossa, and suboccipital craniectomy defects, as well as for extensive temporal bone fractures [29]. The stability of the cement has been confirmed by serial radiographic analyses. Exposure to cerebrospinal fluid did not appear to alter its stability, and pre-existing infection appeared to be the only contraindication for its use. CPC appeared to be superior to acrylic implants for the reconstruction of full-thickness defects of the frontal sinus and frontofacial region by allowing implant osseointegration with improved biocompatibility [30]. Weissman et al. [31] reported successful reconstruction or sealing of defects of the skull base and of facial bones with CPC in 24 patients.

## 7. Conclusion

In July 1996, a CPC consisting of TTCP + DCPA was approved by the Food and Drug Administration for repair of cranial defects in humans, thus becoming the first material of its kind available for clinical use. CPC has become a subject of great interest to many scientists and clinicians worldwide, and several additional CPC products are now commercially available. With continuing improvements in cement properties and understanding of material-tissue interactions under various clinical situations, different CPC formulations with properties optimized for specific clinical applications are being developed.

## Footnotes

1 Certain commercial equipment, instruments, or materials are identified in this paper to foster understanding. Such identification does not imply recommendation or endorsement by the National Institute of Standards and Technology or the ADAHF, nor does it imply that the materials or equipment identified are necessarily the best available for the purpose.
